# The synergy between the insect-inspired claws and adhesive pads increases the attachment ability on various rough surfaces

**DOI:** 10.1038/srep26219

**Published:** 2016-05-20

**Authors:** Yi Song, Zhendong Dai, Zhouyi Wang, Aihong Ji, Stanislav N. Gorb

**Affiliations:** 1 Institute of Bio-inspired Structure and Surface Engineering, Nanjing University of Aeronautics and Astronautics, 29 Yudao Street, 210016, Nanjing, China; 2 College of Mechanical and Electrical Engineering, Nanjing University of Aeronautics and Astronautics, 29 Yudao Street, 210016, Nanjing, China; 3 Department of Functional Morphology and Biomechanics, Kiel University, Am Botanischen Garten 1–9, D-24098 Kiel, Germany

## Abstract

To attach reliably on various inclined rough surfaces, many insects have evolved both claws and adhesive pads on their feet. However, the interaction between these organs still remains unclear. Here we designed an artificial attachment device, which mimics the structure and function of claws and adhesive pads, and tested it on stiff spheres of different dimensions. The results show that the attachment forces of claws decrease with an increase of the sphere radius. The forces may become very strong, when the sphere radius is smaller or comparable to the claw radius, because of the frictional self-lock. On the other hand, adhesive pads generate considerable adhesion on large sphere diameter due to large contact areas. The synergy effect between the claws and adhesive pads leads to much stronger attachment forces, if compared to the action of claw or adhesive pads independently (or even to the sum of both). The results carried out by our insect-inspired artificial attachment device clearly demonstrate why biological evolution employed two attachment organs working in concert. The results may greatly inspire the robot design, to obtain reliable attachment forces on various substrates.

Locomotion is the fundamental property of the majority of animals and has been studied for years^1^,^2^,^3^,^4^. Biologists have attempted to understand structure-function relationships, and to demonstrate how selective pressure shaped evolution of animal locomotion, which is not only efficient and rapid, but also adjustable to various environmental factors. For legged locomotion, the spring-loaded inverted pendulum (SLIP) model and lateral leg-spring (LSL) model were proposed, which clearly describe high efficiency of locomotion by recovering energy during motion^5^,^6^,^7^,^8^. It well known that attachment organs of legged animals are the main way to obtain reaction forces on various substrates. These organs vary not only in the morphologies and distributions, but also in their functional mechanisms^9^,^10^,^11^. They become a key factor for reliable locomotion, especially, when animals move on steep, vertical or even inverted substrates, where adhesive forces are needed to prevent animals from falling down.

Attachment organs can be classified as claws, soft smooth pads and hairy pads^10^,^12^. Claws, observed in most animals, had been experimentally studied and mathematically modelled, especially the relationship between attachment forces of claws, their geometry, and substrate roughness. Previous studies have shown that the stiff cuticle claws of insects attach on rough surfaces by frictional self-lock, in which the friction coefficient between claw tips and the contact surface, the directions of the external forces, acting in contact, are the key factors^13^. Claws may attach reliably only when the mean radii of substrate protrusions are larger than the diameter of claw tips, but work poorly on smooth substrates^13^,^14^,^15^,^16^. To reliably attach on smooth substrate, many animals developed adhesive attachment organs, previously studied in flies^17^, some other insect^18^ and geckos^19^,^20^. Usually, they employ hairy pads and generate adhesion by van der Waals forces^20^,^21^ or capillary forces^17^,^18^,^22^. On the other hand, other animals, such as tree frogs^23^,^24^,^25^, ants^26^,^27^,^28^,^29^, crickets^30^ utilize soft smooth pads and generate adhesive forces through capillary interactions.

However, previous study has shown that the adhesion force generated only by smooth adhesive pads was insufficient to balance the weight of cricket, but it can actually hold an animal on inverted leaves well^30^. We assume that the synergy effect between claws and adhesive pads increase the attachment forces. In order to demonstrate our proposal, we have to study the surface characters of natural substrates and the geometric scale of animal’s attachment organs.

The geometrical scale of surface protrusions of natural substrates, from the nano-crystalline wax structure on the leaf surface to the corrugations of the tree bark, changes 7 to 9 orders of magnitude (Fig. 1A). The range of dimensions of animal attachment pads is also huge, from nano-scale spatulae to macro-scopical big claws (Fig. 1B). Therefore, it can be assumed that insect locomotion must be not only associated with the adhesion on surfaces with nano- to micro- roughness, but also with the attachment abilities on the natural extreme coarse substrates with large protrusions (Fig. 1C). For example, insects bend their tarsi to clasp protrusions and attach to them by both claws and adhesive pads, if the protrusions are comparable to or larger than the tarsus length^31^,^32^. However, definite interactions between insect tarsus and large protrusions are still overlooked in experimental and theoretical studies, since the size of the substrate protrusions (*R*) in previous papers was usually not larger than the mean radius of claw tip (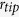) and much smaller than the height of the claw (*h*)^14^,^15^,^16^,^33^,^34^,^35^,^36^.

Here, we proposed a mechanism of synergetic interaction between adhesive pads and claws, which may be responsible for the enhancement of resultant forces between both attachment organs and the substrate. To demonstrate the effect, which would be difficult to demonstrate in natural system, we designed a bio-inspired attachment device by mimicking a system of two claws and adhesive pads. The general idea was to perform the experiment, in which was possible to keep the tested organ and remove the others^14^,^15^, which may heavily influence the performance of tested one. Considering that equivalent spheres were always used to model irregular particles in engineering^37^ and the asperities on sand papers were too small, various steel spheres were applied as the substrates. This approach clearly showed the individual contribution of claws and adhesive pads to the resulting attachment force and a synergy effect, when both types of attachment devices were used simultaneously.

## Results

### The initial friction coefficients and friction angles

**Figure 2:**
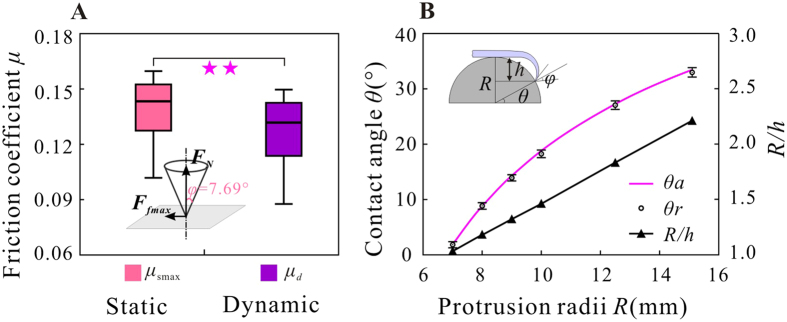
The initial friction coefficients and contact angles. (**A**) The measured maximum of static and dynamic friction coefficients (*μ*_s max_ and *μ*_*d*_) and the corresponding frictional angle *φ*. The significance level **P < 0.05. (**B**) Variations of the analytical contact angle (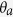), the experimental contact angle (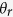), and the relative size (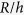) versus protrusion radius *R*.

While in contact with the substrate and sliding along the protrusions, the claws were resisted by the friction forces which were related to the normal force and the friction coefficient between the two claws and the substrates. The maximum static friction coefficient (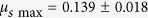, *n* = 36) rather than the dynamic one (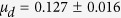, *n* = 36) was taken into consideration (both coefficients were significantly different, *P* = 0.034) (Fig. 2A). The friction angle (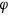) of 7.79° was deduced by using 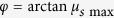.

**Figure 3:**
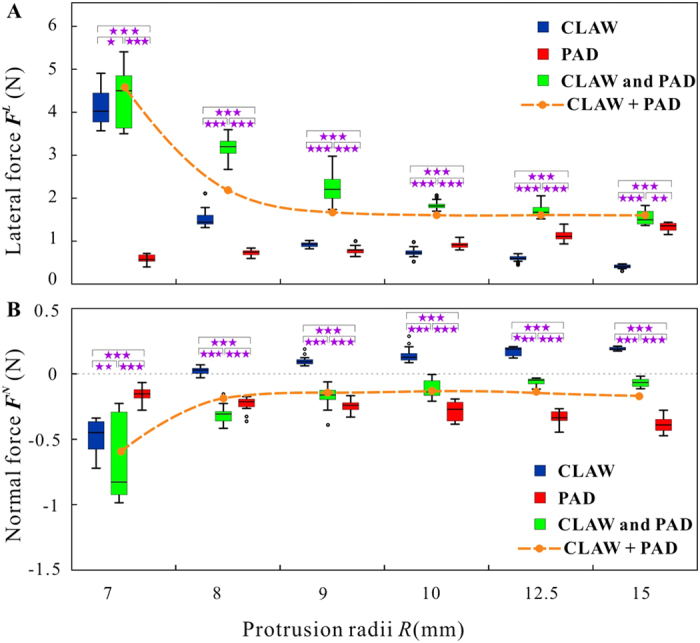
The peak forces on different diameters of spherical protrusions. (**A**) The lateral forces of the claw, adhesive pad and combined device. (**B**) The normal forces of the claw, adhesive pad and combined device. Significance levels: *P > 0.05, **P < 0.05, ***P < 0.001.

To describe the configuration of the attachment device relative to the substrate, a contact angle^13^ (*θ*) was defined as the angle between the horizontal plane and the plane which passes the contact points and the centre of sphere protrusions (Fig. 2B). The real contact angles (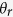) of all trials were also obtained from video images and compared with analytical ones (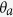). Figure 2B demonstrates that the difference between 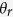 and 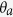 was less than 5%. The contact angles increased with an increase of dimensions of the substrate protrusions. Another parameter (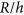), which was defined to characterize the relative size of a protrusion and a claw, increased with increase of the protrusion. As shown in Fig. 3B, the contact angles depend on the relative sizes of claws and protrusions, that is 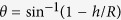. So if the contact angle is less than zero (i.e. 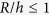 and 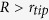 hold), indicating that the protrusions are almost encircled by claw, the claw will never slip off from the protrusions until the claws deform excessively or protrusions break. If 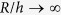, the situation will be same as in the case when the attachment device contacts the plane surface, and the contact angle will be then 90°.

### Attachment on large protrusions

For the convenience of comparisons, three kinds of attachment models were taken into consideration during the experiments. Attachment of (1) the claw (abbreviated as CLAW in Fig. 2) the adhesive pad (abbreviated as PAD), and (3) the combined device (abbreviated as CLAW and PAD) were tested, respectively. Groups of typical reaction force curves, obtained in these three different experiments, are shown in supplementary A (Figs S1, S2 and S3). Variations of the forces in fore-and-aft directions were so insignificant that they were neglected in the result descriptions and further discussions (Fig. 3).

The peak attachment forces of claw on small protrusions (*R* = 7.00 mm) were higher than those on large protrusions (*R* = 10.00 mm). When the claw attached to the protrusions with the radius of 7.00 mm, the peak attachment forces were found to be 4.17 ± 0.47 N (means ± S.D.) in lateral direction and −0.49 ± 0.13N (*n* = 20) in normal direction. However, with an increase of protrusions size, the lateral forces of claw decreased while the normal forces increased. If the radii of protrusions were 8.00 mm, the normal forces were prone to be positive (60%) or negative (40%), while the lateral force was 1.57 ± 0.19N (*n* = 26), which was much less than those on protrusions with the radius of 7.00 mm (*P *< 0.001). When the radius of protrusions was larger than 10 mm, both lateral and normal forces changed only slightly (Fig. 3).

Under similar preloads (*P* > 0.05), the lateral forces of the biomimetic pad varied with protrusion size significantly. When the adhesive pad adhered to protrusions, the lateral forces increased from 0.60 ± 0.11N (*n* = 25, *R* = 7.00 mm) to 1.39 ± 0.13N (*n* = 21, *R* = 15.00 mm). Although the normal forces did not increase as rapidly as the lateral ones, the normal forces (negative) increased from −0.16 ± 0.05N (*n* = 25, *R* = 7.00 mm) to −0.39 ± 0.06N (*n* = 21, *R* = 15.00 mm) with an increase of the sphere diameter (Fig. 3).

The combined device, consisting of two front claws and the adhesive pad, produced significantly higher lateral forces on all large protrusions, which decreased with the size of protrusions. Particularly, when the biomimetic tarsus interacted with protrusions with the radius of 7.00 mm, the lateral force of the combined device was 4.46 ± 0.74N (*n* = 19) and there was no significant difference, if compared with the force of the device consisting just of the claw (*P* = 0.404), but the force was much larger than that measured with the device consisting just of the adhesive pad (*P *< 0.001). However, the normal force (−0.71 ± 0.31N, *n* = 19) was slightly smaller than that of just claw (*P* = 0.024) and all of them were much lower than those obtained on just the adhesive pad (*P *< 0.001). When the radius of protrusions increased to 15.00 mm, the lateral force of the combined device decreased to 1.52 ± 0.16N (*n* = 21), a little larger than that of the adhesive pad (*P* = 0.021) (Fig. 3). These forces were much larger than those of the claw on this kind of protrusions (*P *< 0.001). The normal forces of the combined devices increased with the protrusion size, but kept at the level less than zero: they did not vary, if the radii of protrusions were larger than 9.00 mm.

**Figure 4:**
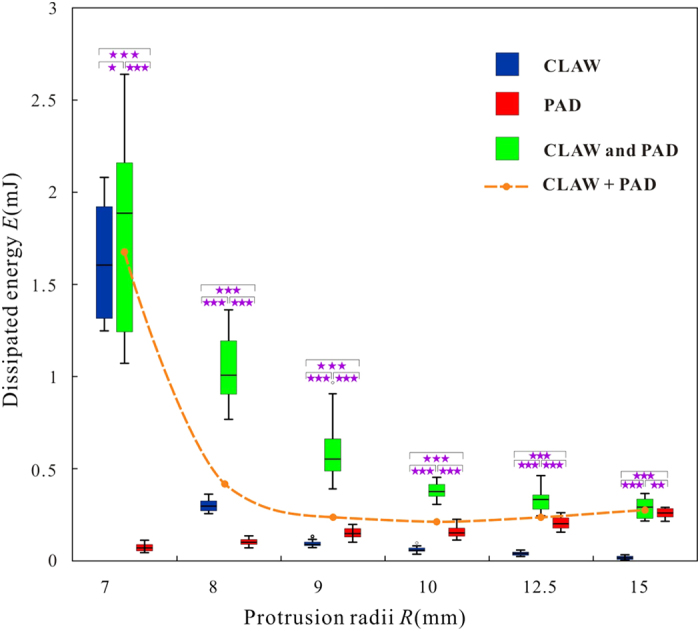
The dissipated energy of the claw, adhesive pad and combined device. Significance levels: *P > 0.05, **P < 0.05, ***P < 0.001.

Figure 4 shows the energy dissipation (*E*) required to break the stable attachments of three tested combinations of attachment devices. Similar to the corresponding peak forces, the dissipated energy of the claw and combined device decreased with the increase of the protrusion size radius. However, the situation was different with the energy of dissipated by the adhesive pad. The dissipated energy of the claw (1.64 ± 0.32 mJ, *n* = 19) and that of the combined device (1.81 ± 0.57 mJ, *n* = 20) on the protrusion with the radius of 7.00 mm was comparable (*P* = 0.611) and was much larger than that measured with just the adhesive pad (0.07 ± 0.02 mJ) (*P *< 0.001). With an increase of protrusion sizes, the dissipated energy of the claw decreased faster than that of the combined devices and approached zero (0.02 ± 0.01 mJ, *n* = 20, *R* = 15.00 mm), if the protrusion was large. The dissipated energy of the combined device converged with that of the adhesive pad with an increased protrusion radius. When the radius was 15.00 mm, dissipated energies were 0.29 ± 0.05 mJ (*n* = 20) for the combined device and 0.26 ± 0.02 mJ (*n* = 21) for the adhesive, respectively (*P* = 0.038).

## Discussion and Conclusions

If the sizes of protrusions are close to the dimension of claw (i.e. 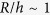), the peak forces and energy dissipation of the claw and the combined device are similar, and much larger than those on other larger protrusions (Figs 3 and 4), indicating that the claw is dominant in generating force on protrusions of such dimensions. We are convinced that the large forces and energy dissipations should be ascribed to the friction locking between claw tips and protrusion surfaces. For example, if the protrusion radius is 7.00 mm, the claw is completely locked by friction because the contact angle (1.85 ± 0.33°) is much smaller than the friction angle (7.79°), resulting in very large reaction forces and energy dissipation. It might be noted that the claw is able to envelope the protrusions and cling steadily, if the protrusion radii are smaller than the claw height (i.e. 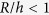), but it may also fail to cling once the protrusion is as small as the claw tips, in which situation our former model^13^ should be taken into consideration. With the increase of protrusion sizes (*R* ≥ 8.00 mm), their contact angles increase and become larger than the friction angle, leading to the failure of the friction locking. The lateral forces of claw decrease, while the normal forces increase with an increase of protrusion radii (Fig. 3), reducing the amount of dissipated energy (Fig. 4). It should be noted that the abovementioned interlocking will be influenced by the friction coefficients between the two materials which determine the friction angles directly. And the claw may generate larger forces in practice if the protrusions are not so regular.

On the other hand, both the peak forces and dissipated energy of the adhesive pad increase with an increasing size of protrusion (Figs 3 and 4), in contrast to the former conclusions that animals’ adhesive pads work poorly on rough surfaces (i.e. the surface asperities are very large)^14^,^15^,^33^,^34^,^35^,^36^. Such an unexpected reversion in force is due to the difference in relative dimension between the tarsus and the substrate. Let us suppose that the adhesive pad contacts with the protrusions elastically and their Young’s moduli and Poisson’s ratios are 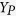, 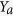, 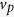 and 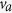, respectively. Then, the radii of contact areas (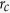) can be calculated using Hertz contact model^38^:


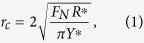


where 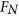 is the normal load, *R*^∗^ is the equivalent radius and *R*^∗^ = *R* holds because the radius of the adhesive pad can be regarded as **∞**; equivalent elastic modulus 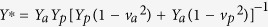 can be regarded as constant. Thus, the contact areas can be written as:


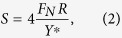


Since the preloads are undifferentiated, the contact areas between the adhesive pad and protrusions are proportional to the radii of protrusions, increasing the forces of adhesive pads because their performances are proportional to their real contact areas with the substrates^39^,^40^.

The peak forces and dissipated energy of the claw and the combined device are similar, but much larger than those of the adhesive pad, when 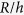 is very close to 1, indicating that the claw is dominant while the adhesive pad is dispensable in this situation. The performance of combined device decreases with an increased protrusion dimensions, however, keeps significantly greater than that of individual claw and that of individual adhesive pad, even greater than their sum (Figs 3 and 4, dashed lines). That is, the forces and energy dissipation of combined device are not just the mathematical sum of those obtained for two structures acting independently, but increased further due of interactions between the two devices.

**Figure 5:**
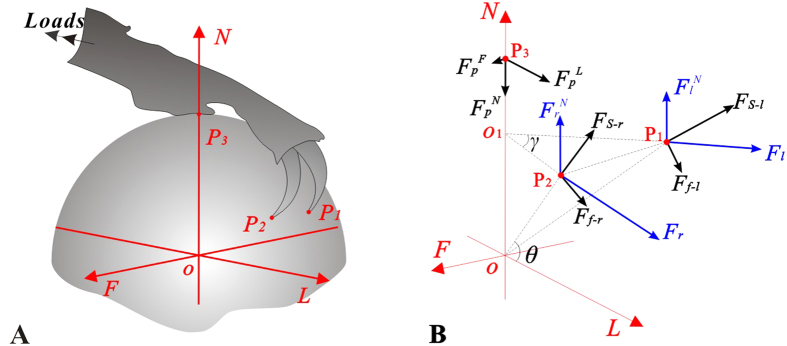
(**A**) 3D contact model. (**B**) The sketch of 3D reaction forces of the biomimetic tarsus devices, where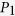, 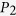 and 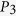 are contact points; 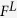 and 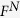 are the maximum external lateral and normal forces; 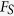 and 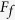 are the local support forces and friction forces at the claw tips. Subscripts ***l*** and ***r*** mean left and right, respectively. ***θ*** and *γ* are the friction angle and the projected angle between the lines running through the claw tips to the sphere centre, respectively.

For the claw which is equipped with adhesive pads, a presentation of the extreme forces can be obtained from a mechanical perspective (Fig. 5):


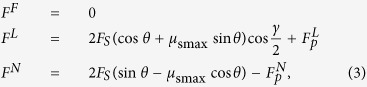


where 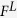 and 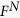 are the maximum external lateral and normal forces, 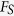 means the local support force at the claw tip, *θ* and *γ* are the contact angle and the projected angle between the lines running through the claw tip to the sphere centre, respectively. Apparently, *θ* and *γ* are *R*-dependent. More detailed information is provided in Supplementary B.

The equation (3) indicates that the increase of contact angle leads to the decrease of the lateral force 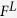, but to the increase of the normal force 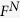. Additionally, the variations of 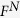 and 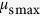 will affect the reaction forces, too. According to the equation (3), when the substrate protrusions are very large (i.e. 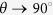), the action of claw will be enhanced significantly, if the pad adheres well. However, if the protrusions are small (i.e. 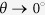), the inter-locking between the claw and the substrates are strong enough to prevent the device detachment even if the adhesive pad work poorly.

It is a common belief that claw works well, if the asperity sizes are large, and that the adhesive pads stick well, if asperity sizes are small. At first glance, the new results seem to contradict this conclusion for we found the claw generate large forces on small protrusions while the adhesive pad adheres well on large protrusions. Actually, as we emphasized above, the relative sizes between the substrate protrusions and the attachment devices (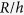) are entirely different from those used in recent experimental and theoretical studies. In the previous reports, the dimensions of substrate protrusions were comparable with the claw tip (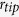) and much smaller than the claw (*h*). Essentially, the large protrusions result in small contact angles here (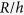) while small asperities result in small contact angles in previous studies^13^ (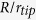).

It can be concluded that when insects attach to the substrate with their tarsi, both claws and adhesive pads participate in force generation, leading to the better performance of the coupled devices. However, the decreasing lateral forces of the claw weaken the lateral performances of coupled device. In the normal direction, adhesive pads mainly contribute to prevent the devices from being detached, especially on very large substrate protrusions. In other words, claws work well through friction locking, if the protrusion sizes are comparable to the claw heights, but possess lower attachment abilities, if the protrusions are significantly larger than the claw heights. On the contrary, the adhesive pad is very effective on large protrusions. The synergetic interactions between claws and adhesive pads not only help insects to stick to small protrusions, but also provide the ability of sticking to protrusions which are much larger than the claw heights, considerably extending the adaptability of combined attachment devices to the natural corrugated substrates.

**Figure 6:**
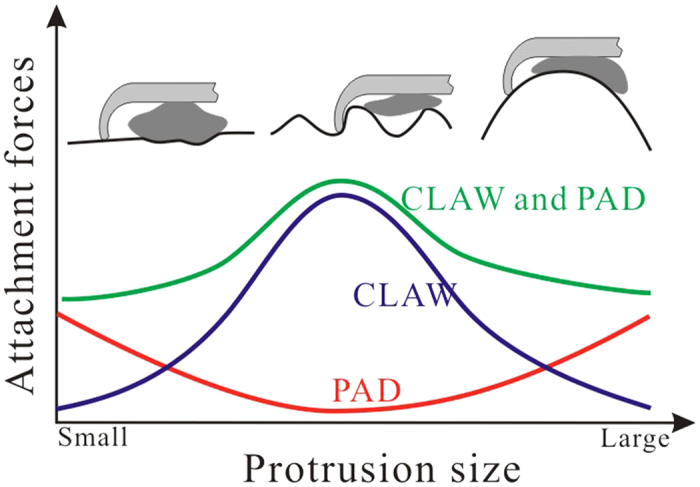
Diagram explaining the effects of protrusion size on the attachment abilities of insect claw and adhesive pad.

Since the asperities, which are larger than the claw length, widely occur in natural substrates, one may suppose that insects apply different attachment modes, in order to cling to or move on these substrates fast and reliably. Considering the results of previous studies, we are convinced that, in the case where the size of protrusions is larger than that of claw tips, but smaller than that of claw heights, animals may use their strong claws to interlock with the protrusions. The considerable interlocking between claw tips and protrusions will guarantee large attachment forces. With the increase of substrate protrusion sizes, the claws with adhesive pads provide much stronger forces. If the substrates are very smooth, the adhesive pads play dominant roles. The increase of adhesive forces by the increase of the contact areas of adhesive pads is probably the widely accepted method of insect adhesion from the point of view of contact mechanics on both very smooth and very rough substrates. Most important of all, the synergic interaction between different attachment organs grantee insects a reliable attachment on various rough surfaces by providing significantly large forces (Fig. 6). Notably, despite of the general synergic principle between claws and adhesive pads to increase the attachment ability on various rough surfaces given by the scale-model, further animal experiments are required to confirm the specific results of different insects attaching on different substrates, because the practical attachment devices of animals are diverse and elaborate.

Recently, significant advances on biomimetic climbing robots were reported^41^. Some attempts have been made to employ claw-like spines and adhesive pads into climbing robots^42^,^43^,^44^,^45^,^46^. Unfortunately, all these attempts are limited by the narrow range of claw dimensions. Attachment devices, combining interlocking claws and adhesive pads can considerably increase the range of surfaces protrusions, which climbing robot can cope with.

## Materials and Methods

As explained above, it is a challenge to study the exact mechanism of coupled attachment devices of insects especially on large substrate protrusions in living animals. We mimicked a typical insect tarsus configuration (mostly corresponding to that of Blattodea, Orthoptera, Mantodea, Phasmatodea, Coleoptera, etc.) with two claws in the front part of the device and an adhesive pad in the hind part. As a substrate, we decided not to use very rough sandpapers (14 grits per inch, with sand particles about 1mm in diameter), because these irregularly shaped and heterogeneously distributed asperities make hard to control the experimental conditions and model the contact. Our device, which consisted of two front claws and a rear-mounted adhesive pad, was manufactured to interact with a series of large spheres mimicking large substrate protrusions. In order to easily fabricate experimental samples and to avoid the fracture of claws and substrate protrusions, we selected steel as the material to manufacture both claws and substrate protrusions.

**Figure 7:**
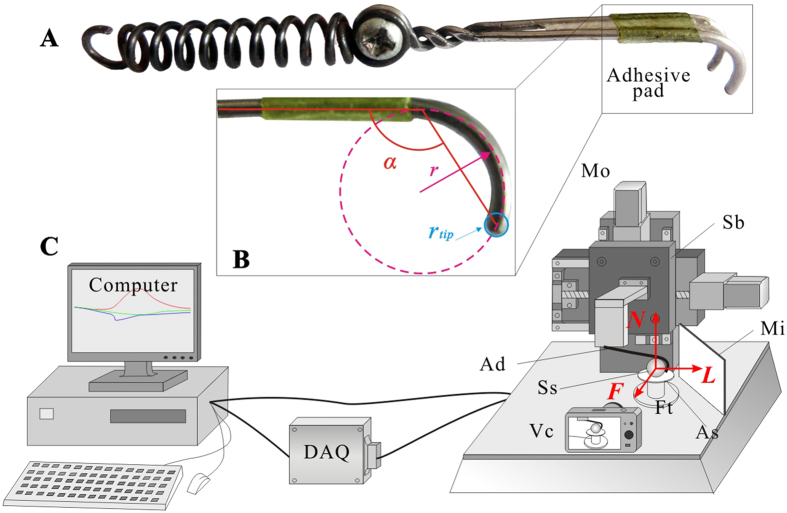
The biomimetic attachment device and measurement system used in the experiments. (**A**) The device consists of two stiff claws, an adhesive pad, and a spring. (**B**) The front view of the claw, 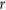 and 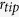 are the radii of claws and claw tips, *α* is the angle between the claw chords and the claw stem. (**C**) The force measurement system consisting of a computer, a data acquisition unit, and a test-bed. The device (Ad) is fixed to the sliding block (Sb) and is moved by the motors (Mo) at constant velocities to interact with the sphere (Ss) which is fixed to the forces transducer (Ft). ***N***, normal direction; ***L***, lateral direction; ***F***, for-aft direction.

As shown in Fig. 7A,B, the biomimetic foot (Ad) is mainly consisted of two stiff claws, an adhesive pad, and a spring. The stiff claws, made of a steel wire with radius of 0.75 mm, had a total length of 48.00 mm. The radius (*r*) of the front claw arcs was 5.02 mm, the gap (***δ***) between two claw tips was 5.12 mm, and the angle (*α*) between the claw chord and stem was 120°. The claw tips were shaped as hemispheres each with radius of 0.75 mm (Fig. 7B). The adhesive pad was mimicked using an adhesive tape, because is capable of bearing shearing and peeling forces. For this purpose, an adhesive film (thickness 0.12 mm, width 9.00 mm) enhanced by a block of soft heat-melt gum was applied to mimic insect adhesives. The adhesive film was replaced after every 5–6 tests to avoid the influence of the tape decay on adhesive force. Since the preload is essential for the proper function of attachment devices^19^, to generate an intimate contact with the substrate, a cylindrical steel spring with length of 65.00 mm and stiffness of 4.50 N/mm was employed and fixed to the end of the force measurement device, with which the biomimetic tarsus was loaded and was able to deflect once the interaction between the tarsus and substrate failed. A series of steel spheres (Ss) with radii ranging from 7.00 mm to 15.00 mm and with surface roughness (*Ra*) less than 0.20 μm were employed to act as the large substrate protrusions to enable interactions with the artificial tarsus and to minimize the influences of microroughness. Pieces of acrylic sheets (As) were glued to the steel spheres so that they can be connected to a multi-axial force sensor that measures the 3D reaction forces of the biomimetic tarsus device under the coordinate system shown in Fig. 7C. The force transducer (Ft) measuring forces and moments separately was calibrated before and after the experiments (Nano 17, ATI, USA).

Three kinds of experiments (individual claw, individual pad and the claw and pad combined) were carried out using a multi-functional test machine (Fig. 7C), where we could move the tarsus with constant velocities in both vertical and horizontal directions. A piece of vertical mirror (Mi) was placed obliquely near to the force sensor so that we could observe the contacts between the claw tips and sphere frontally. To clarify the relative positions of the attachment device and the spheres, a video camera (Vc) was applied to record the experiments at 30 fps.

Apart from the peak forces, the energy (***E***) dissipated to break the stable attachment was also recorded. The dissipated energy was calculated by subtracting the stored energy of spring from the overall energy.


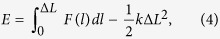


where Δ*L* is the overall elongation of the spring, 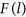 is the force of spring with elongation of *l* and *k* is the spring constant of the spring. Additionally, statistical analyses of peak forces and dissipated energy were carried out using One-Way ANOVA method at the confidence level of 0.05.

## Additional Information

**How to cite this article**: Song, Y. *et al*. The synergy between the insect-inspired claws and adhesive pads increases the attachment ability on various rough surfaces. *Sci. Rep*. **6**, 26219; doi: 10.1038/srep26219 (2016).

## Supplementary Material

Supplementary Information

## Figures and Tables

**Figure 1:**
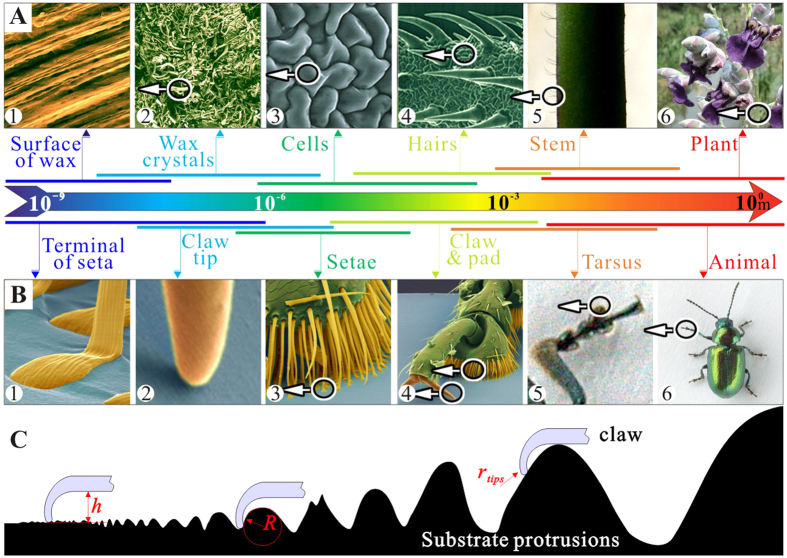
Comparison of different scales of biological devices and natural substrates. (**A**) Surfaces (plants) at different magnifications. The circles and arrows indicate detail views and the color bars indicate possible scale ranges. (**B**) Insect (leaf beetle) with attachment pads and claws at different magnifications. (**C**) Sketch of interactions between insects’ claw and substrate protrusions, where, *h* is the size of the claws, *R* is the mean radius of substrate protrusions and 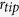 is the radius of claw tips.
