# Supplementary material for: The synergy between the insect-inspired claws and adhesive pads increases the attachment ability on various rough surfaces
Source: Sci Rep. 2016 May 20;6:26219. doi: 10.1038/srep26219 (PMC4873747; doi:10.1038/srep26219)
Supplement: Supplementary Information [file srep26219-s1.pdf]

The synergy between the insect-inspired claws and adhesive pads increases the attachment ability on various rough surfaces

Yi Song[1, 2], Zhendong Dai[1,*] Zhouyi Wang[1], Aihong Ji[1], and Stanislav N Gorb[1, 3,*]

[1] Institute of Bio-inspired Structure and Surface Engineering, Nanjing University of Aeronautics and Astronautics, 29 Yudao Street, 210016, Nanjing, China

[2]College of Mechanical and Electrical Engineering, Nanjing University of Aeronautics and Astronautics, 29 Yudao Street, 210016,Nanjing, China

[3] Department of Functional Morphology and Biomechanics, Kiel University, Am Botanischen Garten 1–9, D-24098 Kiel, Germany

* Corresponding Author: +86 025 84892581, zddai@nuaa.edu.cn, sgorb@zoologie.uni-kiel.de

0. Supplementary A. Forces measured on the three kinds of attachment devices

When the stiff biomimetic tarsus with two claws interacts with the large spherical protrusions, the measured forces are caused by the contact friction between claw tips and sphere surface asperities. Due to the contact friction between claw tips and spheres, the lateral forces increased, while the normal forces decreased continuously until the claw tips slipped off the spheres (Fig.S1). The adhesive pad stuck to the top of protrusions and bore shearing and peeling forces. When peeled vertically, two peak forces were observed, because the cohesive force of the adhesive pad was smaller than the maximum adhesive force. While sheared horizontally, friction forces initially increased and decreased later (Fig.S2). In the tarsus consisting of both claws and adhesive pad, the measured forces curves were slightly similar to those obtained on the stiff claws except for the magnitudes and durations of peak attachment forces. However, the reaction forces did not vary linearly, because of the action of the adhesive pad (Fig.S3). It is worth noting that for all experiments, the reaction forces in fore-and-aft direction were slightly invariable, if compared with normal and lateral forces, indicating that interactions at the two claw tips were almost symmetric.

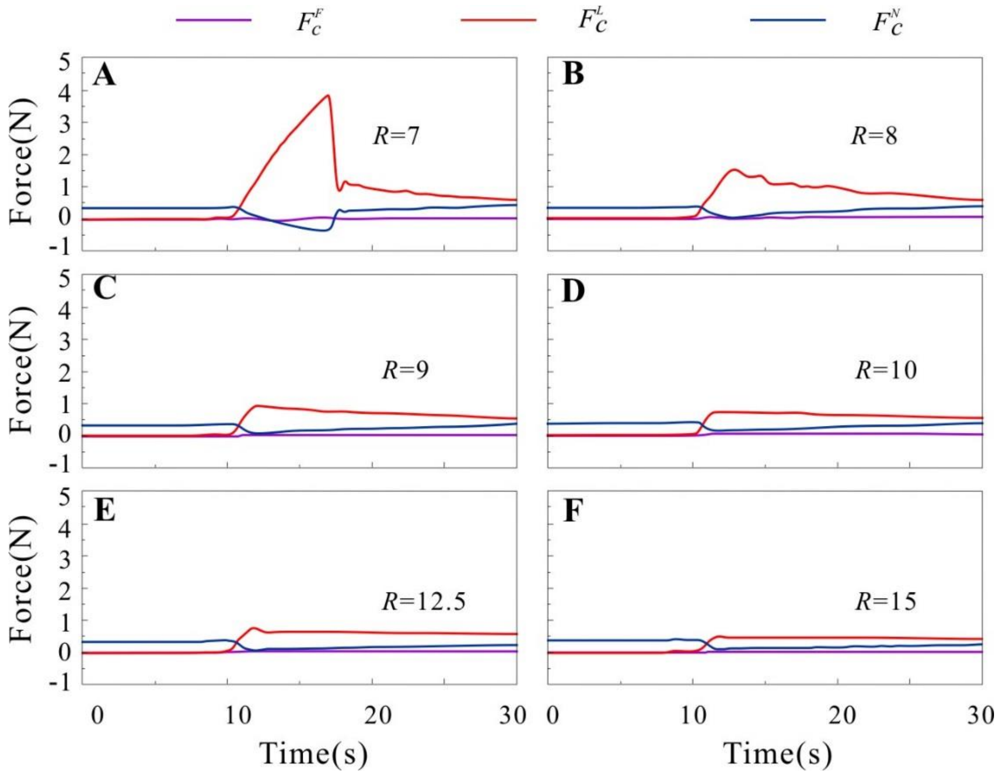

Supplementary Figure S1. The 3-d reaction forces of the stiff claw with two claw tips on sphere protrusions with different radii. A. R = 7.00mm. B. R = 8.00 mm. C. R = 9.00 mm. D. R = 10.00 mm. D. R = 12.50 mm. D. R = 15.00 mm.

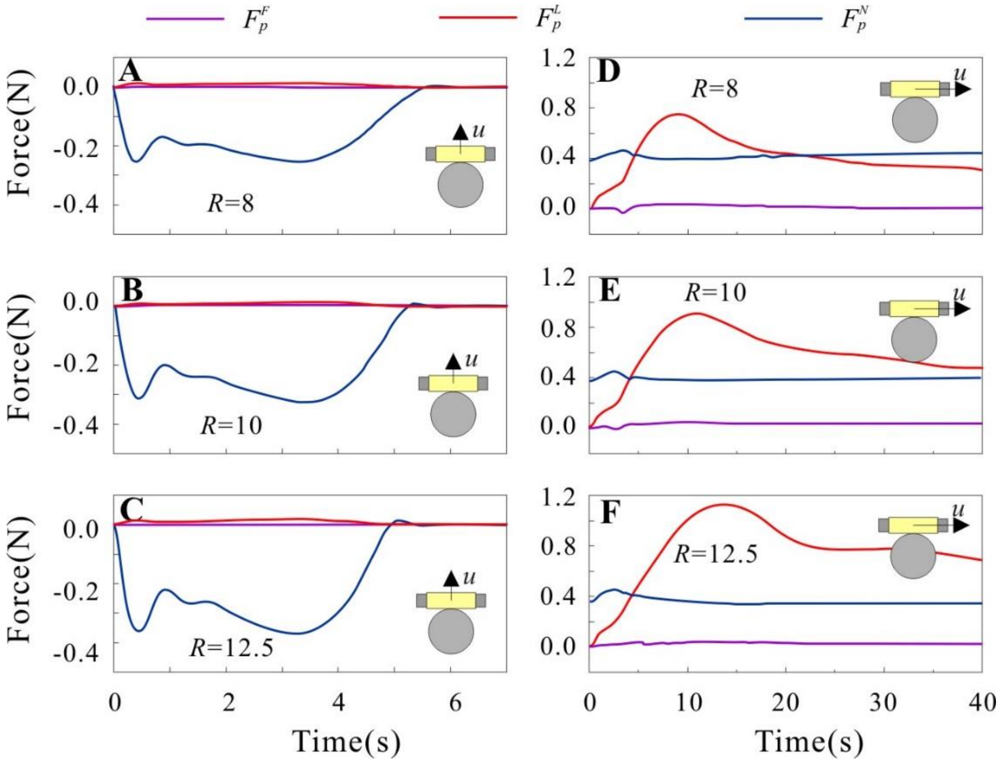

Supplementary Figure S2. The peeling and shearing forces of employed adhesive on spherical protrusions with different radii. A. Peeling, R = 8.00 mm. B. Peeling, R = 10.00 mm. C. Peeling, R = 12.50 mm. D. Shearing, R = 8.00 mm. D. Shearing, R = 10.00 mm. D. Shearing, R = 12.50 mm. The initial pressures are about 0.37 N and there was no significant difference among the initial pressures ($p>0.05$).

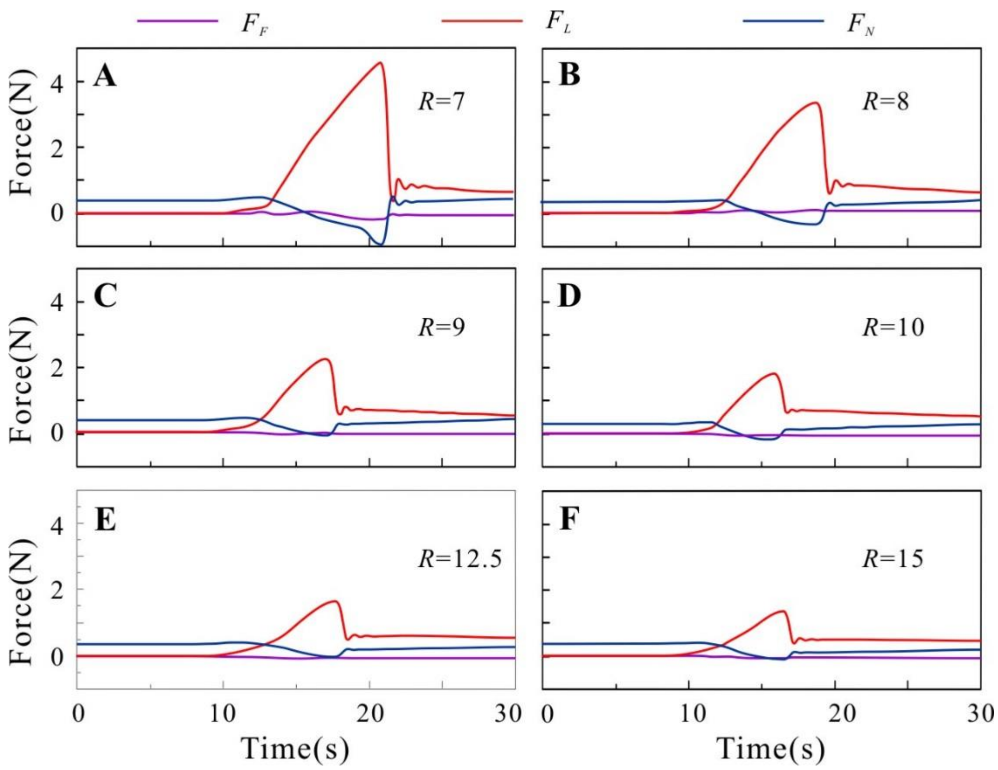

Supplementary Figure S3. The 3-d reaction forces of combined tarsus device consisting of paired claws and adhesive pad on spherical protrusions with different radii. A. R = 7.00 mm. B. R = 8.00 mm. C. R = 9.00 mm. D. R = 10.00 mm. D. R = 12.50 mm. D. R = 15.00 mm.

1. Supplementary B. Model of the contact between attachment devices and large protrusions

To establish the model, four assumptions were made as follow:

(1) When attachment is stable, the configuration of the tarsus device does not change;

(2) Friction F_f and the local support force F_S at claw tips can be described by $F_f = \mu_{smax} \times F_S$;

(3) The adhesive pad sticks to the top of large protrusions.

(4) The two claws are symmetrical and the center of the adhesive pad is coincident with the symmetry plane;

Based on these assumptions, a model was established (Fig.S4 A). Fig.S4 B shows a sketch of the reaction forces.

P_1, P_2 and P_3 are the contact centers of claw tips and adhesive pad, respectively. A coordinate system is set up and shown in Fig.S4. θ is the contact angle and γ is the vertically projected angle between lines (P_1O and P_2O) from the two claw tips to the sphere center. $F_{S\text{-}l}$ and $F_{S\text{-}r}$ are the local support forces at the two claw tips, while $F_{f\text{-}l}$ and $F_{f\text{-}r}$ are the corresponding friction forces; F_p^F, F_p^L and F_p^N are the 3D reaction forces at the adhesive pad which are correlated with the pressure and the pad

itself. Then, the 3D reaction forces at the two tips can be expressed in the coordinate system as follow:

$$\begin{aligned} F_r &= F_{S-r}(\cos\theta + \mu_{s\max}\sin\theta) \\ F_r^N &= F_{S-r}(\sin\theta - \mu_{s\max}\cos\theta) \\ F_l &= F_{S-l}(\cos\theta + \mu_{s\max}\sin\theta) \\ F_l^N &= F_{S-l}(\sin\theta - \mu_{s\max}\cos\theta) \end{aligned} , \tag{S1}$$

Then the resultant forces can be written as

$$\begin{aligned} F_c^F &= (F_r - F_l)\sin\frac{\gamma}{2} \\ F_c^L &= (F_r + F_l)\cos\frac{\gamma}{2} \\ F_c^N &= F_l^N + F_r^N \end{aligned} , \tag{S2}$$

By submitting equation (S1) to (S2), we get:

$$\begin{aligned} F_c^F &= (F_{S-r} - F_{S-l})(\cos\theta + \mu_{s\max}\sin\theta)\sin\frac{\gamma}{2} \\ F_c^L &= (F_{S-r} + F_{S-l})(\cos\theta + \mu_{s\max}\sin\theta)\cos\frac{\gamma}{2} \\ F_c^N &= (F_{S-r} + F_{S-l})(\sin\theta - \mu_{s\max}\cos\theta) \end{aligned} , \tag{S3}$$

γ can be calculated from Fig. S1 B:

$$\sin\frac{\gamma}{2} = \frac{P_1P_2}{2O_1P_1} = \frac{\delta}{2R\cos\theta}, \tag{S4}$$

where δ is the gap between the two tips and R is the radius of protrusions. According to the assumption (1), δ is constant.

To simplify, the local support forces F_{S-r} and F_{S-l} are regarded to be equal, which is in agreement with our experiments results. Thus,

$$\begin{aligned} F_c^F &= 0 \\ F_c^L &= 2F_s(\cos\theta + \mu_{s\max}\sin\theta)\cos\frac{\gamma}{2}, \\ F_c^F &= 2F_s(\sin\theta - \mu_{s\max}\cos\theta) \end{aligned} \tag{S5}$$

According to equation (S5), lateral forces F_c^L will decrease with the increase of contact angle, while the normal forces F_c^N will increase. Obviously, the equation (S5) is similar to our previous Dai-Gorb-Schwarz model[1].

If we take the adhesive pad into consideration, the equation (S5) can be extended as

$$F^F = 0$$

$$F^L = F_p^L + F_c^L = 2F_S(\cos\theta + \mu_{s\max}\sin\theta)\cos\frac{\gamma}{2} + F_p^L\,, \qquad \text{(S6)}$$

$$F^N = F_c^N - F_p^N = 2F_S(\sin\theta - \mu_{s\max}\cos\theta) - F_p^N$$

Equation (S6) indicates that when the substrate protrusions are very large (i e. $\theta \rightarrow 90°$), the action of claws will be enhanced significantly if the pad adheres well. While if the protrusions are small (i e. $\theta \rightarrow 0°$), the inter-locking between claw and substrates are strong enough to prevent the device detach even the adhesive pad work poorly.

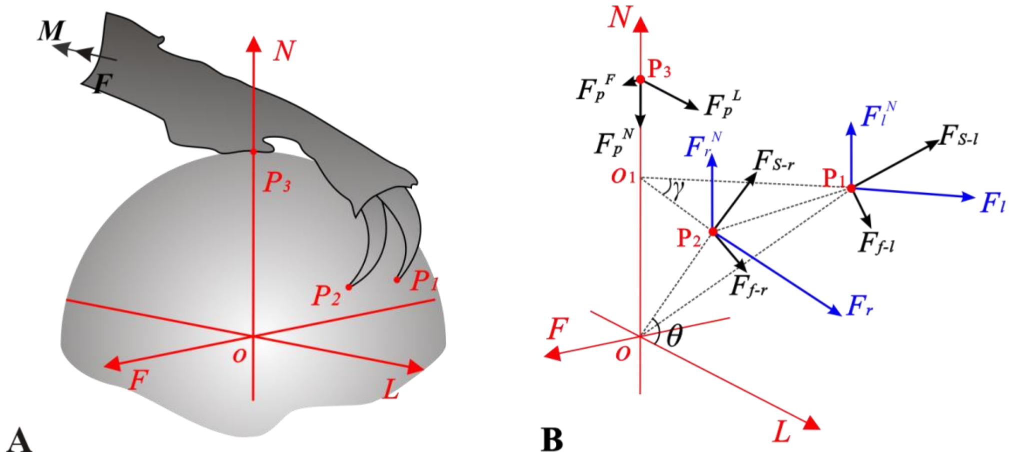

Supplementary Figure S4. A. 3D contact model. B. The sketch of 3D reaction forces of the biomimetic tarsus devices.

References

1. Dai, Z., Gorb, S. N. & Schwarz, U. Roughness-dependent friction force of the tarsal claw system in the beetle *Pachnoda marginata* (Coleoptera, Scarabaeidae). *J. Exp. Biol.* **205,** 2479-2488 (2002).
